# Pharmacokinetic/Pharmacodynamic Target Attainment of Vancomycin, at Three Reported Infusion Modes, for Methicillin-Resistant *Staphylococcus aureus* (MRSA) Bloodstream Infections in Critically Ill Patients: Focus on Novel Infusion Mode

**DOI:** 10.3389/fcimb.2022.874401

**Published:** 2022-07-07

**Authors:** Xiangqing Song, Mi Han

**Affiliations:** Department of Pharmacy, Hunan Cancer Hospital/The Affiliated Cancer Hospital of Xiangya School of Medicine, Central South University, Changsha, China

**Keywords:** vancomycin, methicillin-resistant *Staphylococcus aureus*, pharmacokinetic/pharmacodynamic, continuous infusion, intermittent infusion, optimal infusion

## Abstract

**Objective:**

The study aimed to evaluate and compare the pharmacokinetic/pharmacodynamic (PK/PD) exposure to vancomycin in the novel optimal two-step infusion (OTSI) *vs.* intermittent infusion (II) *vs.* continuous infusion (CI) mode, for MRSA bloodstream infections occurring in critical patients.

**Methods:**

With PK/PD modeling and Monte Carlo simulations, the PK/PD exposure of 15 OTSI, 13 II, and 6 CI regimens for vancomycin, at 1, 2, 3, 4, 5, and 6 g daily dose, was evaluated. Using the Monte Carlo simulations, the vancomycin population PK parameters derived from critical patients, the PD parameter for MRSA isolates [i.e., minimum inhibitory concentration (MIC)], and the dosing parameters of these regimens were integrated into a robust mdel of vancomycin PK/PD index, defined as a ratio of the daily area under the curve (AUC_0–24_) to MIC (i.e., AUC_0–24_/MIC), to estimate the probability of target attainment (PTA) of these regimens against MRSA isolates with an MIC of 0.5, 1, 2, 4, and 8 mg/L in patients with varying renal function. The PTA at an AUC_0–24_/MIC ratio of >400, 400–600, and >600 was estimated. A regimen with a PTA of ≥90% at an AUC_0–24_/MIC ratio of 400–600, which is supposed to maximize both efficacy and safety, was considered optimal.

**Results:**

At the same daily dose, almost only the OTSI regimens showed a PTA of ≥90% at an AUC_0–24_/MIC ratio of 400–600, and this profile seems evident especially in patients with creatinine clearance (*CL*_cr_) of ≥60 ml/min and for isolates with an MIC of ≤2 mg/L. However, for patients with *CL*_cr_ of <60 ml/min and for isolates with an MIC of ≥4 mg/L, the II regimens often displayed a higher or even ≥90% PTA at an AUC_0–24_/MIC ratio of >400 and of >600. The CI regimens frequently afforded a reduced PTA at an AUC_0–24_/MIC ratio of >400 and of >600, regardless of *CL*_cr_ and MIC.

**Conclusions:**

The data indicated that the OTSI regimens allowed preferred PK/PD exposure in terms of both efficacy and safety, and thus should be focused more on, especially in patients with *CL*_cr_ of ≥60 ml/min and for isolates with an MIC of ≤2 mg/L.

## Introduction

Methicillin-resistant *Staphylococcus aureus* (MRSA) is a leading cause of infection worldwide, responsible for a wide range of both hospital and community-acquired infections. The most recent data regarding MRSA incidence, obtained from 85 (44%) of the World Health Organization member states, reported values exceeding 20% in all World Health Organization regions, and even 80% in some countries (Álvarez et al., 2019). The resulting infections due to MRSA often severely limit treatment options because MRSA is often cross-resistant to multiple existing antibiotics.

Some traditional alternatives to vancomycin for MRSA infections, such as trimethoprim–sulfamethoxazole, teicoplanin, daptomycin, linezolid, etc., and new antibiotics in the pipeline for MRSA therapy, such as ceftaroline, ceftobiprole, telavancin, dalbavancin, oritavancin, tedizolid, delafloxacin, radezolid, eravacycline, omadacycline, lefamulin, etc., exhibit good potency for MRSA infections (Lee et al., 2018); however, the traditional agents are unfortunately limited in practice since, compared with vancomycin, these drugs display nonnegligible disadvantages (e.g., inferior efficacy in *S. aureus* endovascular infections for trimethoprim–sulfamethoxazole, less suitability in acute severe infection for teicoplanin, ineffectiveness in pneumonia and central nervous system infections for daptomycin, and bone marrow suppression for linezolid) (Lee et al., 2018). Likewise, those new ones are also limited due to their geographical availability restrictions or unlisting (especially in resource-poor or low-ranking healthcare settings), non-licensing approval, or the lack of high-level evidence for MRSA treatment (Álvarez et al., 2019; Holubar et al., 2020). These predicaments preclude definitive conclusions regarding optimal therapy for such infections and often force clinicians to rely on the suboptimal options derived from high-dose or optimized regimens of existing antimicrobials extrapolated from PK/PD models.

This may be the case for vancomycin. Currently, in MRSA bloodstream infections occurring in critically ill patients, vancomycin is still recommended as a first-line antibiotic by the 2020 vancomycin therapeutic guideline issued by the American Society of Health-System Pharmacists (Rybak et al., 2020b), although the abovementioned potentially effective drugs exist (Lee et al., 2018) and the increase of MRSA isolates with high vancomycin MIC (i.e., ≥1 mg/L) has arisen over the past decade (European Committee on Antimicrobial Susceptibility Testing (EUCAST); The Micron Group). As described in the 2020 vancomycin therapeutic guideline (Rybak et al., 2020b), vancomycin, at an aggressive dosing strategy [i.e., a loading dose of 15 to 20 mg/kg, followed by daily maintenance continuous infusion (CI) of 30 to 40 mg/kg (up to 60 mg/kg)] which is derived from PK/PD prediction and aimed to achieve requisite PK/PD exposure (Rybak et al., 2020b), still remains the standard of care for MRSA infections occurring in critically ill patients, although the approved regimens of 2 g/day vancomycin have little evidence supporting its efficacy for MRSA infections due to isolates with an MIC of even 1 mg/L (Deryke and Alexander, 2009). Understandably, this infusion strategy for maintaining the role of vancomycin in the treatment of MRSA infections seems important.

However, this infusion strategy including CI may be a bit difficult to perform since CI often requires timely therapeutic drug monitoring and monitoring-based dose adjustment to maintain the desired drug exposure. These requirements, however, are often difficult to achieve due to the resistance of the patient to frequent blood sampling and in medical institutions where therapeutic drug monitoring devices are lacking. This results in a common phenomenon that clinicians prefer using the intermittent infusion (II) mode, although the CI mode has the advantages of safety, PK target, and steady-state attainment (Flannery et al., 2020; Yamada et al., 2020). Besides the CI and II modes, a novel infusion mode, i.e., the OTSI mode [a combined infusion mode with an initial loading-rate rapid infusion (LRRI) in the first step and afterwards with immediate low-rate continuous infusion (LRCI) in the second step], for vancomycin, has been recently presented in our previous study, and it showed great attractiveness in terms of PK/PD exposure in non-critically ill patients (Song et al., 2021).

**Figure 1:**
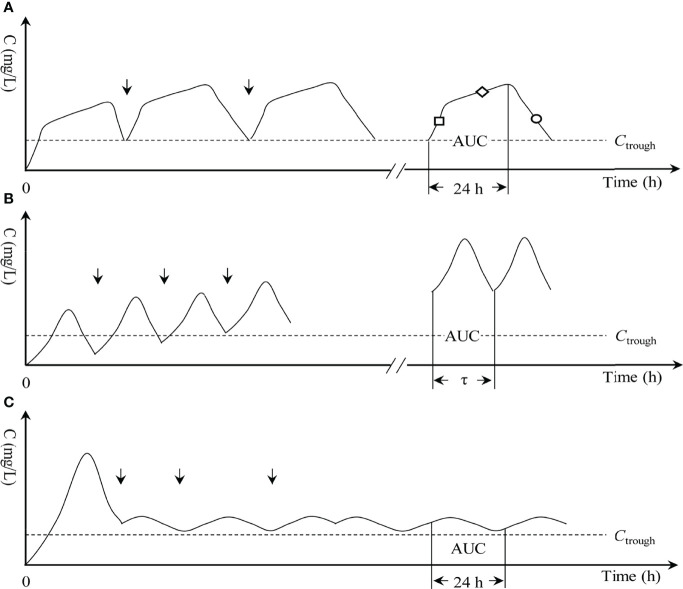
Three infusion modes of vancomycin. **(A)** OTSI, optimal two-step infusion; “□”, loading-rate rapid infusion (LRRI) phase in OTSI mode; “◇”, low-rate continuous infusion (LRCI) phase in OTSI mode; “○”, elimination phase in OTSI mode. **(B)** II, intermittent infusion; τ, dosing interval; and **(C)** CI, continuous infusion. “↓”, start of the dose; AUC, area under the curve; *C*, drug concentration; *C*_trough_, trough concentration.

Proverbially, critically ill patients often show distorted and high PK variability compared with non-critically ill patients (Di Giantomasso et al., 2003; del Mar Fernández de Gatta Garcia et al., 2007; Kees et al., 2011; The Surviving Sepsis Campaign Guidelines Committee including The Pediatric Subgroup* et al., 2013). This phenomenon may result in frequent insufficient vancomycin exposure (Udy et al., 2013; Roberts et al., 2014) and thus increased failure, especially when vancomycin is at an inappropriate infusion mode (since different infusion modes can have a large impact on drug exposure). Therefore, this group and changes in vancomycin exposure due to different infusion modes used in this population should be focused more on. However, it seems that few studies have focused on this issue. Thus, this study aimed to observe the PK/PD exposure of vancomycin at the CI *vs.* II *vs.* OTSI mode (**Figure 1**) for treating MRSA bloodstream infections occurring in critically ill patients to illustrate the concern of which infusion mode has sufficient superiority to resist MRSA infections occurring in critically ill patients, with the concurrent intent of defining optimal dosing regimens for such cases if possible.

## Materials and Methods

### Study Design

With the PK/PD modeling and Monte Carlo simulations, vancomycin population PK models derived from critically ill patients, MIC values fitting those reported in antimicrobial susceptibility testing, and dosing data fitting the clinical administration practice were incorporated as simulated variables into the mathematical model of AUC_0–24_/MIC to observe the probability of target attainment (PTA) provided by vancomycin regimens at the approved PK/PD target, defined as an AUC_0–24_/MIC ratio of 400–600. A 5,000-subject Monte Carlo simulation was performed on AUC_0–24_/MIC under these simulated variables. A regimen with a PTA of ≥90% at an AUC_0–24_/MIC ratio of 400–600 was optimal and acceptable. Based on the PTA obtained, (1) superiority of the three infusion modes and (2) determination of optimal or inferior regimens were further observed.

### Vancomycin PK/PD Target and the Mathematical Models

According to the 2020 vancomycin therapeutic guideline on MRSA infections published by the American Society of Health-System Pharmacists, an AUC_0–24_/MIC target of 400–600 (400 is for the efficacy threshold and 600 is for the safety ceiling) is presently recommended as the primary PK/PD target of vancomycin response considering the efficacy and safety, and traditional trough-only monitoring, with a target of 15–20 mg/L, is no longer recommended (Rybak et al., 2020b). Thus, an AUC_0–24_/MIC ratio of 400–600 was used as the optimal vancomycin PK/PD “efficacy” target in this study. Of note, this AUC_0–24_/MIC target value refers to the total rather than the free AUC_0–24_/MIC value, since they have been interchangeably reported (Rybak et al., 2009). Regarding the mathematical models, under different infusion mode, for calculating the AUC_0–24_/MIC value, they are derived from previous studies as follows.

(I) In the OTSI mode,


$${\text{AUC}}_{0-24}/\text{MIC =}\frac{\begin{array}{l} \frac{\left(24-t_{1}\right)\cdot e^{2CL_{van}/V_{d}\cdot t_{1}}}{CL_{van}}\left[\frac{D_{van}-v_{1}t_{1}}{24-t_{1}}+2v_{1}\left(1-e^{-CL_{van}/V_{d}\cdot t_{1}}\right)\right]\\ +\frac{V_{d}}{CL_{van}^{2}}\cdot \frac{D_{van}-v_{1}t_{1}}{24-t_{1}}\left[e^{CL_{van}/V_{d}\cdot \left(3t_{1}-24\right)}-1\right]+\frac{v_{1}t_{1}\left(2-e^{-CL_{van}/V_{d}\cdot t_{1}}\right)}{CL_{van}}+\frac{V_{d}v_{1}\left(e^{-CL_{van}/V_{d}\cdot t_{1}}-1\right)}{CL_{van}^{2}} \end{array}}{\text{MIC}}$$

(II) In the II mode


$${\text{AUC}}_{0-24}/\text{MIC}=\frac{\frac{24v_{\text{II}}}{\tau \cdot \left(e^{CL_{van}/V_{d}\cdot \tau }-e^{CL_{van}/V_{d}\cdot t_{\inf}}\right)}\left[\frac{t_{\text{inf}}\left(e^{CL_{van}/V_{d}\cdot \tau }-1\right)}{CL_{van}}-\frac{{\left(e^{CL_{van}/V_{d}\cdot t_{\inf}}-1\right)}^{2}}{V_{d}}\right]}{\text{MIC}}$$

(III) In the CI mode,


$${\text{AUC}}_{0-24}/\text{MIC =}\frac{C_{target}^{ss}\times 24}{\text{MIC}}=\frac{24\cdot v_{\text{CI}}}{\text{MIC}\cdot CL_{van}}$$

Note: (1) These equations were built based on steady state and one-compartment intravenous infusion model, (2) Equation 1 and 2 are derived and modified from our previous studies (Song et al., 2021; Song and Wu, 2022) and Equation 3 from the published literatures (Jeurissen et al., 2011; Rybak et al., 2020b).

Where *D*_van_ (mg) is the daily dose, $C_{target}^{ss}$ (mg/L) is the targeted steady-state concentration in the CI mode, AUC_0–24_ (mg·h/L) is the daily area under the concentration-time curve; MIC (mg/L) is the minimum inhibitory concentration; *CL*_van_ (L/h) is the vancomycin clearance; *V*_d_ (L) is the distribution volume; *t*_1_ (h) is the infusion time in LRRI phase of the OTSI mode; *t*_inf_ (h) is the infusion time in the II mode; *v*_1_ (mg/h) is the zero-order infusion rate in LRRI phase of the OTSI mode, calculated as the dose in LRRI phase divided by *t*_1_; *v*_II_ (mg/h) is the zero-order infusion rate in the II mode, calculated as each dose divided by *t*_inf_; *v*_CI_ (mg/h) is the zero-order infusion rate in the CI mode, calculated as *D*_van_ divided by 24 h; *τ* (h) is the dosing interval in the II mode; *e* is the natural constant.

### Vancomycin Population PK Parameter Models

Vancomycin population PK parameters (mainly *CL*_van_ and *V*_d_) models constructed by Roberts et al. (2011), i.e., *CL*_van_ (L/h) = 4.58 × *CL*_cr_ (ml/min)/100, and *V*_d_ (L/kg) = 1.53 × body weight (kg), were used for our analysis since these models (1) revealed good predictive performance for critically ill patients, with minimum mean prediction error of 5.1% [95% confidence interval: −1.2 to 11.4] and minimum median prediction error of −7.5% (95% confidence interval: −34.8 to 28) among six popular vancomycin models in an external validation evaluation (Guo et al., 2019); and (2) were derived from a large cohort study of including 206 intensive care unit patients with various degrees of renal function. Considering renal function changes in critically ill patients and the influence of body weight in *V*_d_, various stages of *CL*_cr_ ranging from 10 to 150 ml/min, with a 30 ml/min increment, were herein simulated, and an adult standard body weight of (mean of 65 kg ± standard deviation of 9.38 kg) (95% confidence interval; 40 to 100) was used for analysis in each stage of *CL*_cr_.

### Simulated Dosing Regimens

Considering the safety, generally per dose of ≤2 g and daily dose of ≤4 g for vancomycin are recommended when vancomycin was used in adults with normal renal function (Lodise et al., 2008; Filippone et al., 2017; Rybak et al., 2020a; USP). However, to predict the interest of increased doses in vancomycin exposure, higher doses of up to 6 g/day were studied in a previous study (del Mar Fernández de Gatta Garcia et al., 2007). Therefore, these doses would be simulated in this study. To accelerate targeted concentration attainment in critically ill patients, a loading dose of vancomycin of 15 to 20 mg/kg when the CI mode for vancomycin was used or even 20 to 35 mg/kg when the II mode for vancomycin was used can be considered according to the 2020 vancomycin therapeutic guideline (Rybak et al., 2020b). Understandably, in the OTSI mode, an initial loading dose of ≥1 g should be thus administered based on 65 kg of standard body weight. Usually, to minimize infusion-related adverse events, vancomycin should be diluted to ≤5 mg/ml and infused over ≥1 h or at a rate of 10 to 15 mg/min (≥1 h per 1 g) according to the 2020 vancomycin therapeutic guideline (Rybak et al., 2020b). Collectively, due to the limit of ≤2 g per dose and of ≥1 h infusion per 1 g, it is understandable that 2–3 h of conventional infusion time for a routine dose of vancomycin is the most frequent. Here, 34 dosing regimens, including 13 II, 15 OTSIs, and 6 CI regimens, are simulated and presented in **Table 1**, along with their dosing parameters.

**Table 1:** Thirty-four simulated dosage regimens and their dosing parameters.

*D*_van_	Infusion mode	Dosing regimens	Dosing parameters
1 g	II	0.25 g q 6 h	*v*_II_, 83–125 mg/h; *t*_inf_, 2–3 h
		0.5 g q 12 h	*v*_II_, 167–250 mg/h; *t*_inf_, 2–3 h
	OTSI	0.5 g LRRI + 0.5 g LRCI	*v*_1_, 167–250 mg/h; *t*_1_, 2–3 h; *v*_2_, 23–24 mg/h; *t*_2_, 21–22 h
	CI	1 g q 24 h	*v*_CI_, 42 mg/h; *t*_CI_, 24 h
2 g	II	0.5 g q 6 h	*v*_II_, 167–250 mg/h; *t*_inf_, 2–3 h
		1 g q 12 h	*v*_II_, 333–500 mg/h; *t*_inf_, 2–3 h
	OTSI	1 g LRRI + 1 g LRCI	*v*_1_, 333–500 mg/h; *t*_1_, 2–3 h; *v*_2_, 45–48 mg/h; *t*_2_, 21–22 h
		1.5 g LRRI + 0.5 g LRCI	*v*_1_, 500–750 mg/h; *t*_1_, 2–3 h; *v*_2_, 23–24 mg/h; *t*_2_, 21–22 h
	CI	2 g q 24 h	*v*_CI_, 83 mg/h; *t*_CI_, 24 h
3 g	II	0.75 g q 6 h	*v*_II_, 250–375 mg/h; *t*_inf_, 2–3 h
		1 g q 8 h	*v*_II_, 333–500 mg/h; *t*_inf_, 2–3 h
		1.5 g q 12 h	*v*_II_, 500–750 mg/h; *t*_inf_, 2–3 h
	OTSI	1 g LRRI +2 g LRCI	*v*_1_, 333–500 mg/h; *t*_1_, 2–3 h; *v*_2_, 91–95 mg/h; *t*_2_, 21–22 h
		1.5 g LRRI +1.5 g LRCI	*v*_1_, 500–750 mg/h; *t*_1_, 2–3 h; *v*_2_, 68–71 mg/h; *t*_2_, 21–22 h
		2 g LRRI + 1 g LRCI	*v*_1_, 667–1,000 mg/h; *t*_1_, 2–3 h; *v*_2_, 45–48 mg/h; *t*_2_, 21–22 h
	CI	3 g q 24 h	*v*_CI_, 125 mg/h; *t*_CI_, 24 h
4 g	II	1 g q 6 h	*v*_II_, 333–500 mg/h; *t*_inf_, 2–3 h
		2 g q 12 h	*v*_II_, 667–1,000 mg/h; *t*_inf_, 2–3 h
	OTSI	1 g LRRI + 3 g LRCI	*v*_1_, 333–500 mg/h; *t*_1_, 2–3 h; *v*_2_, 136–143 mg/h; *t*_2_, 21–22 h
		1.5 g LRRI + 2.5 g LRCI	*v*_1_, 500–750 mg/h; *t*_1_, 2–3 h; *v*_2_, 114–119 mg/h; *t*_2_, 21–22 h
		2 g LRRI + 2 g LRCI	*v*_1_, 667–1,000 mg/h; *t*_1_, 2–3 h; *v*_2_, 91–95 mg/h; *t*_2_, 21–22 h
	CI	4 g q 24 h	*v*_CI_, 167 mg/h; *t*_CI_, 24 h
5 g	II	1.25 g q 6 h	*v*_II_, 417–625 mg/h; *t*_inf_, 2–3 h
		1.67 g q 8 h	*v*_II_, 557–835 mg/h; *t*_inf_, 2–3 h
	OTSI	1 g LRRI + 4 g LRCI	*v*_1_, 333–500 mg/h; *t*_1_, 2–3 h; *v*_2_, 182-190 mg/h; *t*_2_, 21–22 h
		1.5 g LRRI + 3.5 g LRCI	*v*_1_, 500–750 mg/h; *t*_1_, 2–3 h; *v*_2_, 159–167 mg/h; *t*_2_, 21–22 h
		2 g LRRI +3 g LRCI	*v*_1_, 667–1,000 mg/h; *t*_1_, 2–3 h; *v*_2_, 136–143 mg/h; *t*_2_, 21–22 h
	CI	5 g q 24 h	*v*_CI_, 208 mg/h; *t*_CI_, 24 h
6 g	II	1.5 g q 6 h	*v*_II_, 500–750 mg/h; *t*_inf_, 2–3 h
		2 g q 8 h	*v*_II_, 667–1,000 mg/h; *t*_inf_, 2–3 h
	OTSI	1 g LRRI + 5 g LRCI	*v*_1_, 333–500 mg/h; *t*_1_, 2–3 h; *v*_2_, 227–238 mg/h; *t*_2_, 21–22 h
		1.5 g LRRI + 4.5 g LRCI	*v*_1_, 500–750 mg/h; *t*_1_, 2–3 h; *v*_2_, 205–214 mg/h; *t*_2_, 21–22 h
		2 g LRRI + 4 g LRCI	*v*_1_, 667–1,000 mg/h; *t*_1_, 2–3 h; *v*_2_, 182–190 mg/h; *t*_2_, 21–22 h
	CI	6 g q 24 h	*v*_CI_, 250 mg/h; *t*_CI_, 24 h

### Monte Carlo Simulations (Evaluation of Dosage Schedules)

Monte Carlo simulations, performed by the Oracle Crystal Ball software (version 11.1.2; Decisioneering, Inc., Denver, CO, USA) in this study, were used to estimate the PTA of each regimen against isolates with an MIC of 0.5, 1, 2, 4, and 8 mg/L at an AUC_0–24_/MIC target of >400, 400–600, and >600. Regarding the application of the Monte Carlo simulation method in PK/PD study of antibiotics and the principles, software application, and specific implementation of this method, it has been well studied and described elsewhere (Moine et al., 2016; Song and Long, 2018; Song et al., 2021). Briefly, the Monte Carlo simulation method includes the following four steps: (1) setting the distribution patterns of the simulated variables according to their characteristics; (2) setting the confidence interval; (3) incorporating the simulated variables into the mathematical model of AUC_0–24_/MIC; and (4) performing the Monte Carlo simulations on AUC_0–24_/MIC and exporting the PTA values.

Since the Monte Carlo simulation method simulates thousands of patients at given simulated parameters, it is important to acknowledge assumptions made regarding the variability in these parameter estimates. Based on the characteristics of the simulated variables, herein an uniform distribution for *CL*_cr_, *v*_II_, *v*_1_, *v*_2_, *t*_1_, *t*_2_, *t*_inf_, a log-normal distribution for body weight, and a custom distribution for *D*_van_, *v*_CI_, *t*_CI_, and MIC, were assumed. For example, for infected patients with a body weight of 65 ± 9.38 kg and a *CL*_cr_ of 60–90 ml/min due to MRSA isolates with an MIC of 0.5 mg/L, if the II regimen of 0.25 g q 6 h (i.e., *D*_van_ = 1 g) was used, a uniform distribution for *CL*_cr_ in the interval of 60–90 ml/min, for *v*_II_ in the interval of 83–125 mg/h and for *t*_inf_ in the interval of 2–3 h, a log-normal distribution of 65 ± 9.38 kg for body weight, and a custom distribution with a probability of 100% for *D*_van_ at 1 g and for MIC at 0.5 mg/L, were assumed. The confidence interval was set at 95%. With the incorporation of these parameters into the mathematical model of AUC_0–24_/MIC, a 5,000-subject Monte Carlo simulation was performed on AUC_0–24_/MIC to obtain PTA-AUC_0–24_/MIC diagrams with AUC_0–24_/MIC as the abscissa and PTA as the ordinate. The PTA at the AUC_0–24_/MIC target was obtained by assigning the abscissa as the designated target value. In Monte Carlo simulations, the PTA, i.e., the likelihood of a dosage regimen resisting the bacterial isolate at a designated AUC_0–24_/MIC target, is often used to measure the clinical acceptability of a dosage regimen. A regimen with the highest PTA would be optimal as it would provide the highest likelihood of obtaining the targeted exposure for the infectious isolate. Herein, a regimen that maximized the PTA of simulated patients to at least 90% at an AUC_0–24_/MIC ratio of 400–600, which is supposed to maximize both efficacy and safety, was defined as optimal. Regimen that achieved a PTA of ≥90% at an AUC_0–24_/MIC ratio of >400 but <90% (better as low as possible) at an AUC_0–24_/MIC ratio of >600, which is supposed to ensure efficacy but relatively reduce safety, was defined as inferior. Based on the PTA obtained, (1) superiority of the three infusion modes and (2) determination of optimal or inferior regimens were further observed.

## Results

### Probability of Target Attainment

**Figure 2:**
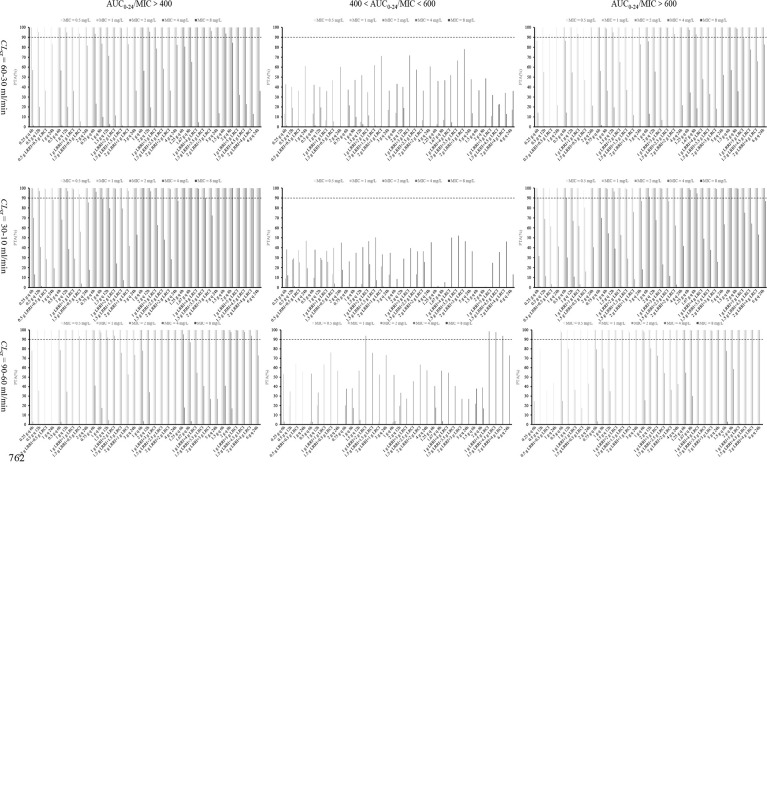
PTA values of 34 dosage regimens for various MICs and *CL*_cr_ at various AUC_0–24_/MIC targets. AUC_0–24_, daily area under the curve; MIC, minimum inhibitory concentration; AUC_0–24_/MIC, ratio of daily area under the curve to minimum inhibitory concentration; *CL*_cr_, creatinine clearance; LRRI, loading-rate rapid infusion in OTSI mode; LRCI, low-rate continuous infusion in OTSI mode.

The PTA of 34 dosage regimens at various *CL*_cr_ and MICs under an AUC_0–24_/MIC ratio of >400, 400–600, and >600 is displayed in **Figure 2**. It can be seen that, under an AUC_0–24_/MIC ratio of 400–600 and for MRSA isolates with an MIC of ≤1 mg/L, only the OTSI regimen of 0.5 g LRRI + 0.5 g LRCI for isolates with an MIC of 0.5 mg/L, 1 g LRRI + 1 g LRCI for isolates with an MIC of 1 mg/L in patients with *CL*_cr_ of >90 ml/min, and the CI regimen of 3 g q 24 h for isolates with an MIC of 1 mg/L in patients with a *CL*_cr_ of 120–150 ml/min, yielded a PTA of ≥90%.

Under an AUC_0–24_/MIC ratio of 400–600 and for MRSA isolates with an MIC of ≥2 mg/L, (I) in patients with a *CL*_cr_ of ≥90 ml/min, all vancomycin regimens at ≤3 g/day failed to achieve a PTA of ≥90%, regardless of the infusion mode. Vancomycin 4 g/day at the OTSI regimen of 1 g LRRI + 3 g LRCI, 1.5 g LRRI + 2.5 g LRCI and 2 g LRRI + 2 g LRCI in patients with *CL*_cr_ of >90 ml/min, 5 g/day at the CI regimen of 5 g q 24 h in patients with *CL*_cr_ of 90–120 ml/min, and 6 g/day at the CI regimen of 6 g q 24 h in patients with *CL*_cr_ of 120–150 ml/min for MRSA isolates with an MIC of 2 mg/L, afforded a PTA of ≥90%. However, no regimen obtained this optimal PTA for those with an MIC of 4 mg/L; (II) in patients with *CL*_cr_ of 60–90 ml/min, only the OTSI regimen of 1 g LRRI + 2 g LRCI for isolates with an MIC of 2 mg/L, 1 g LRRI + 5 g LRCI, 1.5 g LRRI + 4.5 g LRCI and 2 g LRRI + 4 g LRCI for isolates with an MIC of up to 4 mg/L, reached the desired PTA; (III) in patients with *CL*_cr_ of <60 ml/min, no regimen provided a PTA of ≥90%, regardless of the doses, the MICs and infusion mode.

Under an AUC_0–24_/MIC ratio of >400 and >600 and for MRSA isolates with an MIC of ≤1 mg/L, all of the vancomycin regimens at ≥3 g/day achieved a PTA of ≥90% under an AUC_0–24_/MIC ratio of >400, and almost all of these regimens reached this PTA under an AUC_0–24_/MIC ratio of >600, regardless of the infusion mode and *CL*_cr_. No regimen at 1 g/day in patients with *CL*_cr_ of ≥60 ml/min reached a PTA of ≥90% under an AUC_0–24_/MIC ratio of >400. However, the II regimen of 0.25 g q 6 h and 0.5 g q 12 h in patients with *CL*_cr_ of 60–90 ml/min, and the OTSI regimen of 0.5 g LRRI + 0.5 g LRCI in patients with *CL*_cr_ of 30–10 ml/min achieved the requisite PTA under an AUC_0–24_/MIC ratio of >400 but failed under an AUC_0–24_/MIC ratio of >600. All of the vancomycin regimens at 2 g/day obtained a PTA of ≥90% under an AUC_0–24_/MIC ratio of >400 except the II regimen of 1 g q 12 h and the CI regimen of 2 g q 24 h in patients with a *CL*_cr_ of ≥90 ml/min. However, these regimens also achieved this PTA under an AUC_0–24_/MIC ratio of >600 in patients with a *CL*_cr_ of <60 ml/min, except for the CI regimen of 2 g q 24 h.

Under an AUC_0–24_/MIC ratio of >400 and >600 and for MRSA isolates with an MIC of ≥2 mg/L, all vancomycin regimens at ≥4 g/day for isolates with an MIC of 2 mg/L reached a PTA of ≥90% under an AUC_0–24_/MIC ratio of >400 except the II regimens of 1 g q 6 h and 2 g q 12 h and the CI regimens of 4 g q 24 h and 5 g q 24 h in patients with a *CL*_cr_ of ≥90 ml/min. Unexpectedly, these regimens also achieved this PTA under an AUC_0–24_/MIC ratio of >600 in patients with a *CL*_cr_ of <60 ml/min. For isolates with an MIC of 4 mg/L, vancomycin regimens at ≥5 g/day in patients with *CL*_cr_ of only <60 ml/min and those at 6 g/day in patients with *CL*_cr_ of only <90 ml/min yielded the optimal PTA under an AUC_0–24_/MIC ratio of >400, and some of these regimens, such as 1.25 g q 6 h, 1.67 g q 8 h, 1.5 g q 6 h, and 2 g q 8 h in patients with *CL*_cr_ of <60 ml/min provided the desired PTA under an AUC_0–24_/MIC ratio of >600. However, these high-dose regimens in patients with *CL*_cr_ of 30–10 ml/min, achieved a PTA of ≥90% for MRSA isolates with an MIC of up to 8 mg/L under an AUC_0–24_/MIC ratio of even > 600.

### Superiority Comparison of OTSI vs. CI vs. II Mode

It can be seen from **Figure 2** that relative to the II and CI regimens with the same daily dose, only the OTSI regimen reached a PTA of ≥90% under a safe and effective PK/PD target (i.e., an AUC_0–24_/MIC ratio of 400–600), and this profile seems evident especially in patients with a *CL*_cr_ of ≥60 ml/min and for MRSA isolates with an MIC of ≤2 mg/L. These findings suggest that the OTSI mode has certain advantages in terms of efficacy and safety. However, little superiority was shown in patients with a *CL*_cr_ of <60 ml/min and for MRSA isolates with an MIC of ≥4 mg/L. In contrast, the II regimens for these patients and these isolates often displayed a superior and even ≥90% PTA under an AUC_0–24_/MIC ratio of >400 relative to the OTSI and CI regimens, implying that the II mode exhibited an extrapolated increase in terms of efficacy. However, these regimens also displayed a higher or even ≥90% PTA under an AUC_0–24_/MIC ratio of >600, implying that the II mode exhibited a concomitant increase in terms of safety risk. Interestingly, the CI regimens frequently afforded a reduced PTA under an AUC_0-24_/MIC ratio of >400 and of >600, regardless of the *CL*_cr_ and MICs. This implied that the CI mode presented reduced efficacy and safety risk. Contrastively, the OTSI mode allowed the optimal PK/PD target attainment with both efficacy and safety.

### Determination of Optimal or Inferior Regimens

In the absence of better options for treating MRSA bloodstream infections occurring in critically ill patients, this study summarizes the optimal or inferior vancomycin regimens that we considered may be effective based on the PTA obtained. **Table 2** displays these regimens for such infections occurring in critically ill patients with different *CL*_cr_ and caused by MRSA isolates with different MICs.

**Table 2:** The optimal or inferior vancomycin regimens that the present study considered may be effective for the treatment of MRSA bloodstream infections occurring in critically ill patients, at different *CL*_cr_ and MICs.

*CL*_cr_ (ml/min)	Rank^b^	Potentially effective regimens at various MICs (mg/L)^a^				
		0.5	1	2	4	8
150–120	Optimal	0.5 g LRRI + 0.5 g LRCI	–	1 g LRRI + 3 g LRCI	NA	NA
	Second-line	–	1 g LRRI + 1 g LRCI	1.5 g LRRI + 2.5 g LRCI 2 g LRRI + 2 g LRCI	NA	NA
	Third-line	–	1.5 g LRRI + 0.5 g LRCI 3 g q 24 h	NA	NA	NA
120–90	Optimal	0.5 g LRRI + 0.5 g LRCI	1 g LRRI + 1 g LRCI	1 g LRRI + 3 g LRCI	NA	NA
	Second-line	0.25 g q 6 h	1.5 g LRRI + 0.5 g LRCI	1.5 g LRRI + 2.5 g LRCI 2 g LRRI + 2 g LRCI 1 g q 6 h	NA	NA
	Third-line	–	0.5 g q 6 h	5 g q 24 h	NA	NA
90–60	Optimal	–	–	1 g LRRI + 2 g LRCI	2 g LRRI + 4 g LRCI	NA
	Second-line	0.5 g LRRI + 0.5 g LRCI	1.5 g LRRI + 0.5 g LRCI	1.5 g q 12 h	1.5 g LRRI + 4.5 g LRCI 1 g LRRI + 5 g LRCI 1.25 g q 6 h	NA
	Third-line	1 g q 24 h 0.5 g q 12 h	1 g LRRI + 1 g LRCI 2 g q 24 h 1 g q 12 h	1 g q 8 h 0.75 g q 6 h	1.5 g q 6 h 2 g q 8 h	NA
60–30	Optimal	NA	–	–	–	–
	Second-line	NA	0.5 g q 12 h	1 g q 12 h	0.75 g q 6 h	1.5 g q 6 h
	Third-line	NA	0.25 g q 6 h	0.5 g q 6 h	NA	NA
30–10	Optimal	NA	–	–	–	–
	Second-line	NA	0.5 g LRRI + 0.5 g LRCI	0.5 g q 12 h	1 g q 12 h	0.75 g q 6 h
	Third-line	NA	NA	NA	NA	NA

a NA, Not applicable; “–”, Not available.

b The optimal regimen was determined by such a regimen: (1) it has a PTA of ≥90% at an AUC_0–24_/MIC ratio of only 400–600 and a minimum one at an AUC_0–24_/MIC ratio of >600 and (2) it has the lowest daily dose. A second-line regimen was determined by such a regimen: (1) it has a PTA of ≥90% at an AUC_0–24_/MIC ratio of only 400–600 but a higher one at an AUC_0–24_/MIC ratio of >600 relative to the optimal regimen, and (2) has the same daily doses as the optimal regimen; or such a regimen: (1) it has a PTA of ≥90% at an AUC_0–24_/MIC ratio of only >400 and a minimum one at an AUC_0–24_/MIC ratio of >600, and (2) has the same or reduced daily doses as the optimal regimen. The third-line regimen was determined by such a regimen: (1) it has a PTA of ≥90% at an AUC_0–24_/MIC ratio of only >400 but a higher one at an AUC_0–24_/MIC ratio of >600 relative to the second-line regimen and (2) it has the same daily doses as the second-line regimen; or such a regimen: compared with the second-line regimen, it is the optimal regimen in the next daily dose.

## Discussion

To our knowledge, this is the first study to evaluate the PK/PD exposure of vancomycin at CI, II, and OTSI modes, for MRSA bloodstream infections occurring in critically ill patients with various *CL*_cr_ and caused by MRSA isolates with different MICs. The data here supported that in critically ill patients: (1) the II mode displayed competitiveness for MRSA isolates with an MIC of ≥4 mg/L in efficacy but also increased risk in safety; (2) the CI mode presented no superiority in efficacy but reduced risk in safety; and (3) the OTSI mode showed certain advantages for MRSA isolates with an MIC of ≤2 mg/L in both efficacy and safety. The data, included in **Table 2**, can better inform tentative vancomycin regimens for treating MRSA infections occurring in critically ill patients in the absence of better options for such infections.

In critically ill patients, more current studies on vancomycin II *vs.* CI have focused mainly on the comparison in terms of the efficacy and safety of vancomycin (Vuagnat et al., 2004; Hutschala et al., 2009; Akers et al., 2012; Saugel et al., 2013; Schmelzer et al., 2013; Hanrahan et al., 2014; Tafelski et al., 2015; Bissell et al., 2020). However, comparative studies on the outcomes of vancomycin II vs. CI against MRSA isolates with a specific MIC are still scarce. A study (Akers et al., 2012) compared the clinical outcomes of vancomycin at the II regimen of 1 g q 8 h and the CI regimen of 3 g/day used in critically ill patients and indicated that an average of 2.3 g/day vancomycin, at the II regimens or with the II mode, fell below a trough of 15 mg/L more than half of the time. It implied that this dosage was insufficient and poor efficacy was thus obtained. However, this study did not provide the outcomes of vancomycin II vs. CI against MRSA isolates with a specific MIC. Theoretically, a vancomycin level of 25 mg/L for increasing MICs (≥1 mg/L) in *S. aureus* seems more appropriate (Wang et al., 2006), and would maintain an AUC_0–24_/MIC ratio of ≥400 against isolates with an MIC of 1.5 mg/L, assuming constant vancomycin levels over 24 h. To achieve this level, vancomycin 3 g/day, if with the CI mode, should be required when MRSA infections occur in critically ill patients with a *CL*_cr_ of ≥120 ml/min (Jeurissen et al., 2011). Similarly, the data presented in the present study suggested that in such critically ill patients, 3 g/day vancomycin, with the CI mode, obtained a PTA of ≥90% for isolates with an MIC of 1 mg/L under an AUC_0–24_/MIC ratio of >400; however, whether this regimen (i.e., 3 g q 24 h) can achieve this optimal PTA for an MIC of 1.5 mg/L has not been studied. However, for critically ill patients with a *CL*_cr_ of <60 ml/min, this regimen achieved a PTA of ≥90% for isolates with an MIC of up to 2 mg/L under an AUC_0–24_/MIC ratio of >400. Another study, which was aimed to evaluate the clinical outcomes of vancomycin CI vs. II in the treatment of severe staphylococcal infections, observed vancomycin response on 40 randomly selected strains (of which 18 in II group and 22 in CI group) with an MIC of ≤2 mg/L (Wysocki et al., 2001). In this study, although 31 cases were treated successfully, the kinds of MICs and the II or CI regimens were not reported. The current lack of comparative studies on clinical outcomes of vancomycin II *vs.* CI against isolates with a specific MIC has impeded the comparison of the superiority of the II mode *vs.* CI mode, especially against those with high MICs. However, this study indicated that the II mode for vancomycin displayed its competitiveness against isolates with an MIC of ≥4 mg/L relative to the CI mode and may therefore be a preferred dosing strategy in such cases when alternatives to vancomycin are unavailable.

Of interest, in most current clinical studies in vancomycin, one challenge when evaluating its clinical outcomes is preferring reporting of concentration—rather than AUC-indicated vancomycin exposure, regardless of vancomycin in the CI (usually reporting steady-state concentration) or II (usually reporting trough concentration) mode. However, these reported concentration values may not be adequate surrogates for AUC-indicated efficacy exposure in critically ill patients (Turner et al., 2018), as the AUC is the integrated quantity of cumulative drug exposure (i.e., the serum drug concentration-time curve over a defined interval), while the trough represents a single exposure point at the end of the dosing interval. Moreover, in clinical practice, monitoring of trough concentrations will be often be translated into the achievement of one specific minimum daily AUC value (Rybak et al., 2020b). Although trough-only monitoring is practical, the potential limitations surrounding the practice suggest that trough monitoring is insufficient to guide vancomycin dosing in all patients (Rybak et al., 2020b).

Nevertheless, in some simulated studies, AUC- or AUC/MIC-based vancomycin exposure was partially observed. A Monte Carlo simulation study conducted by del Mar Fernández de Gatta Garcia et al. (2007) indicated that in critically ill patients with a mean *CL*_cr_ of 65.5 ml/min, 3–4 g/day vancomycin, if with the II mode, against isolates with an MIC of 1 mg/L, would be required to provide a PTA of 90% under an AUC_0–24_/MIC ratio of 400, thus questioning the standard regimens of 2 g/day vancomycin II against isolates with such MICs. However, this study suggested that in critically ill patients with a *CL*_cr_ of 60–90 ml/min, 2 g/day vancomycin at the II regimen of 0.5 g q 6 h or 1 g q 12 h and 3–4 g/day vancomycin at a II regimen of 1 g q 8 h or 2 g q 12 h against isolates with an MIC of 1 and 2 mg/L, respectively, is sufficient to achieve a PTA of 90%. Another Monte Carlo simulation study, conducted by Setiawan et al., (2019), on vancomycin exposure against MRSA, reported that in patients with *CL*_cr_ of 60–120 ml/min, 2 g/day vancomycin at the II regimen of 1 g q 12 h, 3 g/day vancomycin at a II regimen of 1 g q 8 h or 1.5 g q 12 h and 4 g/day vancomycin at a II regimen of 1 g q 6 h or 2 g q 12 h provided a PTA of 100% for an MIC of 0.5 mg/L at an AUC_0–24_/MIC ratio of 400. However, if with II mode 3 g/day vancomycin for an MIC of 1 mg/L and 4 g/day vancomycin for an MIC of 1.5 mg/L should be required for attaining a PTA of ≥90%, thus doubting the approved 2 g/day vancomycin, at the II regimens, against isolates with such MICs. Consistently, these regimens displayed similar outcomes in the present study, especially for patients with a *CL*_cr_ of 120–150 ml/min. Inconsistently, however, 2 g/day vancomycin at the II regimen of 0.5 g q 6 h for an MIC of 1 mg/L and 4 g/day vancomycin at a II regimen of 1 g q 6 h for an MIC of up to 2 mg/L exhibited a PTA of nearly 100% in patients with *CL*_cr_ of 60–120 ml/min. Discordance of the results between these and this study may be due to the used vancomycin PK models. The study by del Mar Fernández de Gatta Garcia et al. (2007) used a PK model of *CL*_van_ (ml/min/kg) = 0.660 − 0.016 × age(years) − 0.006 × Acute Physiology and Chronic Health Evaluation System score + 0.380 × serum albumin (g/dl) + 0.562 × *CL*_cr_ (ml/min/kg) and the study by Setiawan et al., (2019) used a PK model of *CL*_van_ (L/h) = 0.0444 × *CL*_cr_ (ml/min) to predict PTA of vancomycin regimens. Herein, a PK model of *CL*_van_ (L/h) = 4.58 × *CL*_cr_ (ml/min)/100 was used. Understandably, using different PK models may result in different predicted results. We believe that the data herein are believable because the chosen vancomycin PK models were considered to have broad applicability for critical population since these models were derived from a large cohort study of including 206 intensive care unit patients with various degree of renal function, and revealed good predictive performance for critically ill patients (Guo et al., 2019).

Eguchi et al. proposed the strategy of optimal two-step infusion therapy and established the corresponding PK/PD index model in 2010 (Eguchi et al., 2010). However, this strategy was for time-dependent antibiotics. OTSI mode is a new infusion mode recently proposed and built for vancomycin, a concentration-dependent antibiotic, in our previous study (Song et al., 2021). Currently, little clinical data on this infusion mode exist. However, its theoretical superiority was exhibited both in efficacy and safety, and both in critically ill patients and non-critically ill patients. The present study indicated that at the same daily dose, almost only the OTSI regimens showed a PTA of ≥90% at an AUC_0–24_/MIC ratio of 400–600, especially for patients with a *CL*_cr_ of ≥60 ml/min and against isolates with an MIC of ≤2 mg/L. It implies that this infusion mode maximizes both the efficacy and safety of vancomycin. Although the II regimens displayed a higher or even ≥90% PTA at an elevated MIC of ≥4 mg/L under an AUC_0–24_/MIC ratio of >400, they also obtained this PTA under an AUC_0–24_/MIC ratio of >600. It suggested that the II mode presented concomitant increased efficacy and safety risks. The CI regimens did not afford a higher PTA at an AUC_0–24_/MIC ratio of >400 but obtained a reduced PTA at an AUC_0–24_/MIC ratio of >600, implying that no increase in efficacy but a lower risk of safety was displayed. This outcome in the CI mode for vancomycin is consistent with that obtained by previous studies (Van Herendael et al., 2012; Schlobohm et al., 2021). In non-critically ill patients, 2 g/day vancomycin at the OTSI regimen of 1.95 g LRRI + 0.05 g LRCI and 4 g/day vancomycin at the OTSI regimen of 2 g LRRI + 2 g LRCI for an MIC of up to 2 mg/L and 4 mg/L, respectively, achieved a PTA of ≥90% at an AUC_0–24_/MIC ratio of 400, but failed if at the II regimens (Song et al., 2021). This suggests the superiority of OTSI mode in improving efficacy. However, reduced PK/PD target attainment is still observed in critically ill patients compared with non-critically ill patients. This may be due to the distorted vancomycin PK variability in critically ill patients, as demonstrated in previous studies (Di Giantomasso et al., 2003; del Mar Fernández de Gatta Garcia et al., 2007; Kees et al., 2011; The Surviving Sepsis Campaign Guidelines Committee including The Pediatric Subgroup* et al., 2013).

The theoretical superiority of the OTSI mode in efficacy and safety is understandable because, according to the design of this mode, it cannot only rapidly reach the initial drug concentration of the multifold MIC but also reduce the fluctuation of peak and trough concentration. This not only rapidly inhibits the target strains but also alleviates the toxic side effects caused by high peak concentrations and the bacterial resistance caused by low trough concentrations. With increasing antibiotic resistance, this mode of vancomycin would be helpful in the clinic, particularly considering delays in the development of new alternatives and a lack of better treatment options. However, due to the lack of clinical data on this infusion mode, these theoretical advantages still lack experimental validation.

For MRSA infections occurring in critically ill patients, **Table 2** summarizes some potentially effective vancomycin regimens based on our analysis, and these regimens can better inform us of tentative treatment in the absence of better options for such infections. Of note, despite this significant case of Monte Carlo simulation prediction, these potentially effective regimens based on Monte Carlo simulations cannot be considered certainly effective given the difference in action profiles among antibiotics and in resistance mechanisms among bacteria. Additionally, the modification of vancomycin delivery in severe infections may not be sufficient, by itself, to change the clinical outcome for critically ill patients. Moreover, risk factors associated with a novel vancomycin delivery, such as safety, may be points of concern. However, all of the dosage regimens here were set under a safe PK/PD index and dosing parameters (including dose, infusion rate or time, etc.). Understandably, this novel OTSI mode should be safe.

The main limitation of this study lies in its theoretical nature. This study relied on the PTA to evaluate the efficacy, which has potential limitations as it is only a probability value and therefore lacks sufficient power to detect clinical outcomes. However, Monte Carlo simulation-based feasibility for optimizing exposure to improve antimicrobial effectiveness has been expounded and applied in OPTAMA studies (Kuti and Nicolau, 2005) and PTA-indicated theoretical efficacy has been demonstrated by Eguchi et al. in an *in vitro* PD model study on meropenem against *P. aeruginosa*, in which *in vitro* viable cell counts of *P. aeruginosa* strain were used as a measure for the *in vitro* bactericidal activity of meropenem (Eguchi et al., 2010). Thus, we believe that our approach is appropriate since the vancomycin population PK model used here was derived from critically ill patients and PK variability was taken into account; the PD target was adopted from the 2020 vancomycin therapeutic guideline; the MIC values corresponded to those reported in antimicrobial susceptibility testing; and the emulational dosing parameters were close to clinical practice. Therefore, the results on vancomycin dosage could be applied if patient and pathogen populations match those considered here. If this was not the case, the same methodological procedure could be followed, but the actual PK (relationship between *CL*_van_ and *CL*_cr_, *V*_d_, and body weight due to patient variables) and PD modeling (MIC values) would have to be used. Nevertheless, large clinical trials would be of benefit to determine the competency of CI, II, and OTSI regimens in critically ill patients. Also, therapeutic drug monitoring for vancomycin might be necessary considering its high PK variability in critically ill patients, especially involving the efficacy and safety at a high dose.

## Conclusions

Critically ill patients manifest physiology that is unlikely to be encountered in an ambulatory or ward-based environment. Due to the distorted PK profile of vancomycin in these patients, the II and CI modes for vancomycin used in these groups may be unable to achieve an optimal balance in terms of both efficacy and safety. Based on the PK/PD end points, the data presented here show that the OTSI mode for vancomycin allows optimal PK/PD target attainment in terms of both efficacy and safety and it should be therefore focused more on when vancomycin is used for treating MRSA bloodstream infections occurring in these groups. However, large trials are needed to validate these regimens and their clinical implications, especially involving the balance of efficacy and nephrotoxicity at a high dose. Therefore, we agree with the opinion that therapeutic drug monitoring for vancomycin might be necessary considering the high PK variability of vancomycin in critically ill patients.

## Data Availability Statement

The original contributions presented in the study are included in the article/supplementary material. Further inquiries can be directed to the corresponding author.

## Ethics Statement

Ethical approval/written informed consent was not required for the study of animals/human participants in accordance with the local legislation and institutional requirements.

## Author Contributions

XS performed the modeling simulations and wrote the manuscript. HM conceptualized and supervised the manuscript. All authors listed have made a substantial, direct, and intellectual contribution to the work and approved it for publication.

## Conflict of Interest

The authors declare that the research was conducted in the absence of any commercial or financial relationships that could be construed as a potential conflict of interest.

## Publisher’s Note

All claims expressed in this article are solely those of the authors and do not necessarily represent those of their affiliated organizations, or those of the publisher, the editors and the reviewers. Any product that may be evaluated in this article, or claim that may be made by its manufacturer, is not guaranteed or endorsed by the publisher.
